# Speeding Access to Vaccines and Medicines in Low- and Middle-Income Countries: A Case for Change and a Framework for Optimized Product Market Authorization

**DOI:** 10.1371/journal.pone.0166515

**Published:** 2016-11-16

**Authors:** Vincent Ahonkhai, Samuel F. Martins, Alexandre Portet, Murray Lumpkin, Dan Hartman

**Affiliations:** Integrated Development – Global Health, Bill & Melinda Gates Foundation, Seattle, Washington, United States of America; Western Sydney University, AUSTRALIA

## Abstract

**Background:**

The United Nations Millennium Development Goals galvanized global efforts to alleviate suffering of the world’s poorest people through unprecedented public-private partnerships. Donor aid agencies have demonstrably saved millions of lives that might otherwise have been lost to disease through increased access to quality-assured vaccines and medicines. Yet, the introduction of these health interventions in low- and middle-income countries (LMICs) continues to face a time lag due to factors which remain poorly understood.

**Methods and Findings:**

A recurring theme from our partnership engagements was that an optimized regulatory process would contribute to improved access to quality health products. Therefore, we investigated the current system for medicine and vaccine registration in LMICs as part of our comprehensive regulatory strategy. Here, we report a fact base of the registration timelines for vaccines and drugs used to treat certain communicable diseases in LMICs. We worked with a broad set of stakeholders, including the World Health Organization’s prequalification team, national regulatory authorities, manufacturers, procurers, and other experts, and collected data on the timelines between first submission and last approval of applications for product registration sub-Saharan Africa. We focused on countries with the highest burden of communicable disease and the greatest need for the products studied. The data showed a typical lag of 4 to 7 years between the first regulatory submission which was usually to a regulatory agency in a high-income country, and the final approval in Sub-Saharan Africa. Two of the three typical registration steps which products undergo before delivery in the countries involve lengthy timelines. Failure to leverage or rely on the findings from reviews already performed by competent regulatory authorities, disparate requirements for product approval by the countries, and lengthy timelines by manufacturers to respond to regulatory queries were key underlying factors for the delays.

**Conclusions:**

We propose a series of measures which we developed in close collaboration with key stakeholders that could be taken to reduce registration time and to make safe, effective medicines more quickly available in countries where they are most needed. Many of these recommendations are being implemented by the responsible stakeholders, including the WHO prequalification team and the national regulatory authorities in Sub-Saharan Africa. Those efforts will be the focus of subsequent publications by the pertinent groups.

## Introduction

The United Nations Millennium Development Goals have galvanized global efforts to alleviate suffering of the world’s poorest people [1]. With these major global challenges as a reference, new and unprecedented public-private partnerships were formed. One of the most effective of these coalitions, the GAVI Alliance, has since its inception in 2000 provided vaccines to approximately 440 million of the world’s poorest children [2,3]. Similarly, a focused campaign led by The Global Fund to Fight AIDS, Tuberculosis and Malaria (Global Fund), increased funding for malaria elimination by more than 18-fold between 2000 and 2011, contributing to a significant reduction in the incidence of malaria in 34 endemic countries [4]. The continuing efforts of these aid organizations and others such as UNITAID and the U.S. President's Emergency Plan for AIDS Relief (PEPFAR) demonstrate that dramatic results can be achieved by increasing healthcare access [5]. Looking to the future, global product development partnerships have accelerated to the point that new interventions for diseases afflicting primarily low- and middle-income countries (LMICs) are ready or nearly ready for use [6].

But reaching populations in need continues to prove challenging as we at the Bill & Melinda Gates Foundation (BMGF) frequently observe working with partners to fulfil our mission. The challenges point to a high degree of complexity in the interplay of stakeholders in the global regulatory and delivery systems. The key stakeholders include global and regional pharmaceutical companies, non-governmental organizations, national medical product regulatory agencies (NRAs), ministries of health and others who make product utilization recommendations. In particular, regulatory and procurement requirements vary widely between countries, creating a system with many inefficiencies and redundancies, only a few of which have been adequately documented [7].

Our intuition was that the resultant drug lag [8] should be addressed in order to improve health for millions in underserved nations. Therefore, we worked with several partners to investigate timelines for the global registration process for both vaccines and medicines intended to prevent or treat communicable diseases. At the project inception in 2012, data on the subject were not available in the public domain except for regulatory agencies in high-income countries (HIC).

We set out to develop the fact base to enable us answer the following key questions:

- What are the registration pathways for global health products reaching LMICs?
- How much time does each step take in the pathways and what is the cumulative end-to-end time?
- Who are the stakeholders involved and what are their responsibilities?
- Where are the biggest bottlenecks, challenges, and gaps?
- What solution options can be synthesized from the fact base?

Here, we present a review of the current LMIC registration system for biopharmaceutical products and propose a framework that could help reduce product registration time. The reduced time could in turn accelerate access to health products and ultimately reduce morbidity and mortality consistent with our philanthropic mission.

## Methods

From November 2012 through June 2013, we investigated the time between regulatory submission and approval for vaccines and drugs introduced into LMIC markets. We focused on

1. the registration steps for global health products which were made affordable through UN agency procurement or donor aid and
2. considered to have met safety, efficacy and quality standards through the World Health Organization (WHO) prequalification system or
3. the United States Food and Drug Administration (FDA) and European Medicines Agency (EMA) approval systems times, and
4. full end-to-end timelines for the same product category

For our data collection and stakeholder interviews we contracted a management consulting firm; however, the data interpretation and conclusions were our responsibility. We discussed our findings with the groups that provided the data and with experts. The stakeholders involved include pharmaceutical product developers and manufacturers, regulators from high-income countries (FDA, EMA) and from Sub-Saharan African countries the WHO PQ team and procurers (Global Fund, GAVI, UNITAID, UNICEF). We segmented vaccines and medicines based on their registration paths, identifying two key segments for each: products that first registered with a high-income (Stringent) Regulatory Authority ("SRA first"), and those that first registered with a LMIC NRA (“Non-SRA first”).

No human or animal research was involved in our study and no personal or identifying information such as address or birth dates were collected during our fact finding process.

## Results

The criteria for our analysis: vaccines and medicines for the prevention or treatment of communicable disease which were in circulation in LMICs through procurement by UN agencies or PEPFAR. The products had to have been prequalified by the WHO or registered by an SRA or non-SRA between 2009 and 2012. There was a modest number of products that was in the LMICs through the private market of multinational pharmaceutical companies.

### Registering medical products in the global marketplace, a three-step process

**Fig 1:**
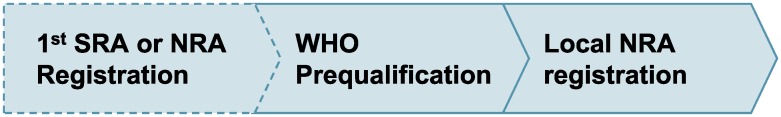
Three-step registration process to developing countries. For medicines, the “1^st^ Registration” is not required as a pre-requisite for WHO-PQ.

New vaccines and drugs undergo a step-wise series of preclinical and clinical testing to ensure safety and efficacy, and must meet appropriate manufacturing quality standard before they can be registered for sale. This product development process can be lengthy, often stretching over more than a decade. For the purpose of this study, our timeline begins after the development process is complete and the manufacturing company makes its first registration submission to a regulatory authority. The UN agencies-procured products which we reviewed typically undergo a three-step registration process involving two categories of regulatory authorities and the WHO-PQ (Fig 1).

#### Step 1. First registration

Once the development process is complete, initial product registration historically has occurred in the country where the product is manufactured. Among high-income countries, the regulation of pharmaceutical products is relatively uniform, in part due to the International Conference on Harmonisation of Technical Requirements for Registration of Pharmaceuticals for Human Use (ICH), a collaboration initiated in 1990 between the US, EU, and Japan aimed at adhering to a uniform set of scientific and technical standards. Generally, countries that fully adhere to ICH standards are termed SRAs and they play major roles in global health as the first regulator of almost all novel drugs and most vaccines used in developing countries. However, a steadily increasing number of generic versions of innovative products is now first registered by non-SRAs, such as the Central Drugs Standard Control Organization of India and the China Food and Drug Administration (CFDA).

#### Step 2. Quality assurance required by procurement agencies

International aid agencies which fund the purchase of vaccines and essential medicines for LMICs such as GAVI, UNICEF and the Global Fund, typically require a separate quality assurance assessment as a prerequisite for procurement. This assessment for “prequalification (PQ) for potential tendering” is conducted by WHO in its role as an independent, science-based evaluator ensuring that healthcare products maintain international standards for quality, safety, and efficacy. Steps 1 and 2 can be accomplished together when a product is first registered with an SRA and a procurer of that product accepts SRA registration for quality assurance.

WHO PQ for vaccines was established in 1996 and for medicines in 2001 and both involve an assessment of the performance of the products, benefit-to-risk ratio for LMIC populations and the suitability of the product for conditions of intended use (programmatic suitability). To date, the WHO PQ has prequalified more than 500 medicines, 120 vaccines, 50 in-vitro diagnostics and 2 male circumcision devices [9,10]. In recent years, the program has expanded its scope and influence, with WHO PQ becoming an essential imprimatur for any company seeking to sell medicines in bulk to any LMIC-focused aid agency. Given its expanded and influential role, PQ serves as a focal point for interactions among regulatory authorities from high-income countries and LMICs NRAs and manufacturers. Thus, the efficiency of PQ assessments serves to increase the availability of affordable medicines in LMICs.

#### Step 3. Registration with national regulatory authorities (NRAs)

As in high-income countries, NRAs in LMICs have a mandate to ensure the safety, efficacy, and quality of products delivered to their populations, therefore, they require local registration of products [11,12,13]. This constitutes the third of the 3-step process. These national authorities in theory have an important role given that they understand the health needs and challenges of providing medicines to their citizens [14]. However, the mandatory individual review by multiple countries, each with its own regulatory authority, processes, and capability challenges leads to increased complexity and long product approval timelines.

### Lengthy reviews delay availability of medicines

The products included in our analysis consisted of 205 vaccines and medicines which were WHO prequalified from 2009 to 2012. A total of 44 products underwent the full 3 registration steps. Seventy (70) were approved by FDA and EMA, and Swissmedic. All products ultimately reached LMICs after 2 or 3 steps.

**Fig 2:**
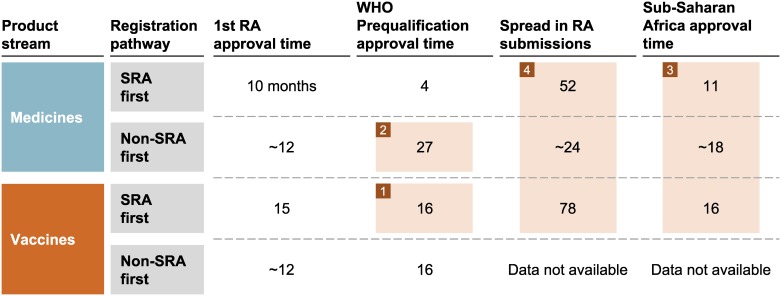
Registration Timelines (median in months) for Global Health Products (herein defined as products eligible for WHO Prequalification) Distributed in Sub-Saharan Africa (as of December 2012).

The data, summarized in Fig 2, shows that the time between first regulatory authority submission for a given drug or vaccine to its registration in the last (by disease burden) of 20 Sub-Saharan Africa countries was typically between 4 and 7 years. There are many factors responsible for this lengthy timeline, e.g. redundancy across steps since there was no leveraging of the technical reviews already performed by competent bodies, inefficiencies in the regulatory processes themselves-, and failure of manufacturers to meet the international standards required by WHO-PQ. We identified the following four main factors and the timelines resulting from them.

#### 1) SRA-approved vaccines took a median of 16 months to complete the WHO PQ process

In contrast, the PQ time for drugs that have been SRA approved was 4 months because the medicines PQ team did not repeat many of the regulatory activities already performed by SRAs (e.g. scientific assessments and inspection of manufacturing facilities). The root causes of the longer vaccines PQ timelines are:

1. The WHO PQ team conducted many of the same review activities for vaccines, such as manufacturing site inspections, for all vaccines, regardless of whether such activities had already been conducted by an SRA.
2. Manufacturers of some vaccines that were approved first by an SRA and distributed in high-income countries were missing important clinical data relevant to the target LMIC population. For example, in the case of vaccines, clinical trials were not conducted in accord with the WHO immunization schedules nor with co-administration of other vaccines used in the National Immunization Programs (NIP) of LMICs. Also, stability profiles were often not in compliance with the NIP conditions and the finished product presentations were sometimes not consistent with the necessary programmatic suitability for LMIC use.
3. Manufacturers also contribute substantially to the delay in the PQ process for vaccines as a result of their slow response to WHO questions; 9 months of the 16-month vaccine PQ review (over 50%) is accounted for by time spent waiting for manufacturer responses.

#### 2) Generic medicines from emerging markets required more than 2 years (median of 27 months) to complete the PQ process

The standards for the registration of generics (e.g. in India or China) were often less stringent than the ICH standards that PQ requires. Therefore, additional time was often required for manufacturers from those countries to raise the standards of their submissions to meet the PQ requirements. A recent study of generic product dossiers submitted to WHO PQ found that most contained significant deficiencies, either in safety or quality data [15]. In addition, as with vaccine reviews, significant time (16 months) was spent waiting for manufacturer responses to requests for improvements of deficiencies, typically resulting from lack of prioritization and capacity constraints on the manufacturer’s end.

Interestingly, generics manufacturers also register products in high-income countries such as the US. However, they usually meet the required ICH quality standards in those countries because they prioritize such commercial market approval over the UN Agency-procured markets.

#### 3) Products first registered by an SRA or PQ process took an additional 1–2 years to receive NRA approval in Sub-Saharan Africa

NRAs often repeated assessments of quality, safety, and efficacy already performed by SRAs or PQ, such as dossier review, product sample testing, and manufacturing site inspections. NRAs sometimes explained this redundancy as a requirement to ensure that SRA and PQ-approved products are the same as they are receiving in their countries. Though a legitimate concern, redundant assessments may not be the solution.

As noted by others, we observed that African nations with the greatest burden of disease also have the most resource limited NRAs [6]. While little systematic study has been conducted to document the current status of NRAs in Africa, an assessment by the WHO offers some information. WHO assessment teams interviewed NRA regulators in 26 African countries and found across the board weak management structures and processes, severe lack of qualified staff, and few available resources [15]. Detailed records, including the length of individual review processes, are typically not available.

#### 4) Manufacturers often spread submission of new products for Sub-Saharan Africa NRA approval over several years

We identified several potential root causes of this time spread. First, as mentioned earlier, large multi-national manufacturers typically did not prioritize early registration and introduction of their novel products into low-income countries. This is due to limited commercial potential in most of those countries.

Additionally, varying requirements and legislative frameworks in low-income countries limit the ability of manufacturers to submit a single dossier concurrently to those countries. The enormous resources required to prepare unique submissions for each country and respond to questions from each individual NRA may have exacerbated this spread. As a result, some countries experience long waits before they even receive application dossiers for review.

**Fig 3:**
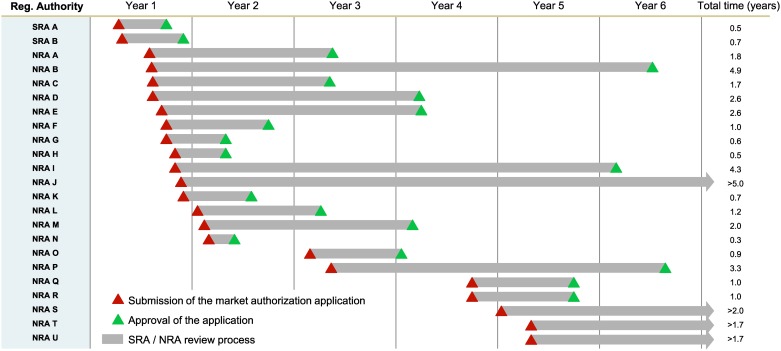
Registration Application Submissions and Approval in SSA for an anonymized antiretroviral drug, demonstrating 1) the variance in approval timelines across SSA countries and 2) the spread in manufacturer submissions. Red triangles represent the moment of dossier submission to the SRA or NRA, green triangles the market authorization approval by the NRA, and the grey arrows the review process.

Fig 3 provides a representative example of the gap between the first regulatory submission and the last in anonymized SSA countries as well as approval times for an anti-retroviral drug. We collected similar data on dozens of drugs and vaccines that follow a similar pattern. First registration was typically followed by quality assurance procedures conducted by WHO PQ. Over subsequent years, the product would undergo evaluation procedures in perhaps 20 or more additional African countries. As a result, the last country on the registration list was waiting up to 7 years for access to a vaccine or medicine that is available in surrounding countries.

The time between the first and last market registration application spanned a median of 78 months for the eight vaccines considered in our analysis. The time span for registration of new drugs was also lengthy, with a median of 52 months.

In our interviews with pharmaceutical company representatives, we learned that the sequence and timing of regulatory submissions often depended on factors such as the issuance of the Certificate of Pharmaceutical Products (CPP). This is a document which is required by NRAs in LMIC as evidence that the product has indeed been registered in a high-income country. Although NRAs require that the CPP be submitted with dossier, in many cases the CPP was not available until much later.

In other instances, manufacturers may not have demonstrated the performance of their product in the underlying health conditions affecting some African countries. Such was the case with a tuberculosis treatment that could not be used in Africa because it had not been tested in HIV-positive patients. HIV may be common in TB patients in Sub-Saharan Africa [6].

## Discussion

### Framework for an optimized market authorization system

Based on the data presented and discussions with key stakeholders, we evaluated scenarios and developed a set of potential actions to address the root causes of the key problems identified in global health product registration system. These proposed approaches [Table 1] are designed to build upon elements of the existing system that are working well, while introducing efficiencies that improve the system for all stakeholders.

**Table 1:** A Framework for Optimized Market Authorization System for Products in LMICs.

Strategy axis	Key underlying initiatives
Focus on value-added activities	Increase focus on value-added activities by all stakeholders, minimizing repetition (e.g. re-inspection of a site already inspected by an SRA, repeat scientific assessments) and relying on work products of trusted regulatory bodies and WHO-PQ. Maximize efficiency of WHO-PQ programme Expand WHO accelerated NRA review programmes
Decrease complexity	Harmonize regulatory standards and requirements and implement regionally across groups of countries
Improve manufacturer inputs	Manufacturers and Regulatory Authorities enforce WHO-PQ or international ICH standards

#### Focus on value-added activities

Given that the 3-step registration process we described usually starts with a comprehensive assessment by well-resourced regulatory agencies such as the FDA and EMA, we believe that any subsequent assessment ought to avoid repeating those assessments. In this regard, PQ and NRAs in Sub-Saharan Africa can improve system efficiency by leveraging SRA assessments and focusing on activities that add value or fill any gaps in the assurance of safety, efficacy or quality mentioned earlier. Eliminating mandatory laboratory testing of samples or GMP inspections could shorten registration time and save resources. In addition, dedicating more resources for post-market safety monitoring and pharmacovigilance is a suitable approach to ensure satisfactory performance of products.

Such reliance on the work products of trusted counterpart agencies to inform one’s own regulatory decision making is already being implemented by several regulatory authorities. Examples include the various GMP reliance programs by countries which are members of Pharmaceutical Inspection Cooperation Scheme (PIC/S), and abbreviated marketing application reviews by the NRAs of Switzerland, Singapore and Mexico when certain products have already been authorized for marketing by EMA or FDA.

Cooperative activities such as these should be further expanded and made standard operating procedure.

#### Decrease complexity

Optimizing the fragmented regulatory system must become a global health priority. In our analysis, we found many complexities in the current system such as disparate NRA standards and requirements in LMICs which led to additional work or duplicative work by manufacturers when filing applications in different African countries. Given the limited commercial returns in LMICs, it makes sense to eliminate duplicative efforts and, adopt a common set of technical requirements for product registration. The resource toll in time and cost engendered in re-writing and managing applications from one country to another remains a disincentive for manufacturers. Concern about more consistent and optimized access to quality medicines in Europe was one of the strongest reasons for developing the unified approach to pharmaceutical regulation that today exists in the European Union [16]. Efforts to apply mutual recognition procedures should be encouraged and expanded in other parts of the world.

New initiatives to address complexity are now emerging partly as a result of the data presented herein. Development of uniform standards among neighbouring countries with closely tied economic interests (if the countries are willing to work together) provides one promising approach. In 2012, the regulatory agencies of Burundi, Kenya, Rwanda, Tanzania, Uganda, and Zanzibar, launched an African Medicine Regulatory Harmonisation (AMRH) pilot program [17]. The AMRH initiative is a public-private partnership of an African agency (the New Partnership for Africa’s Development, NEPAD), the WHO, and donor organizations. It aims to improve the continent’s regulatory systems by moving from a country-focused approach to simplified approaches through regional collaboration and harmonization of regulatory standards. The creation of the AMRH has provided a platform to mitigate African regulatory capacity constraints and reduce dossier submission challenges. AMRH implementation has now expanded to other African Regional Economic Communities in West (ECOWAS—Economic Community of West African States), Southern (SADC—Southern African Development Community), and Central (ECCAS—Economic Community of Central African States) regions. These initiatives are providing further opportunities to address the complexity problem. Similar regionalization is also occurring in other parts of the world, including the Pan American Health Network for Drug Regulatory Harmonization [18].

#### Improve manufacturer inputs

Our data showed that manufacturers often did not devote adequate resources to obtaining LMIC product registrations in a timely manner. A recent study of generic product dossiers submitted to WHO PQ found that most contained significant deficiencies, either in safety or quality data [19]. In many cases, product registration dossiers submitted by generic product manufacturers included insufficient data to demonstrate that a generic product submitted for registration is bioequivalent to its reference or originator product. Regulatory agencies have an obligation to insist on critical international standards such as bioequivalence when approving a generic medicine. If countries that account for the majority of generic products submitted for WHO PQ enforced internationally recognised standards that ensure bioequivalence and product manufacturing quality, the PQ process could be accelerated.

Decreasing complexity and optimizing the existing regulatory infrastructure would also help alleviate some of the regulatory burden on manufacturers. However, if manufacturers improved the initial quality of submitted registration dossiers further efficiencies could be generated within the current system. Manufacturers could contribute to faster product registration if they commit to regulatory planning during product development and engage with regulators and WHO early in the course of product development They should also commit to promptly responding to regulatory queries on their dossiers.

### Early impact

On a positive note, among the approaches we have proposed to address delayed access to quality pharmaceutical products in LMICs, we would like to acknowledge some examples of past successes and more recent promising initiatives.

A past example illustrating how reliance and cooperation can speed access to health products is the N. meningiditis serogroup A polysaccharide-tetanus toxoid conjugate vaccine (PsA–TT), MenAfriVac. Manufactured by the Serum Institute of India (Pune, India) the vaccine was first licensed in 2009 by the Indian NRA with technical support from Health Canada and it subsequently gained WHO prequalification. The WHO PQ team then facilitated rapid approvals in Sub-Saharan Africa NRAs in 2010 by providing the NRAs access to WHO’s assessment reports, including results of laboratory testing and site inspections. The subsequent fast introduction of the vaccine and rapid immunization of more than 215 million people resulted in a huge drop in meningitis cases that has been well documented [20].

In another recent example of the impact of leveraging and reliance between organizations, WHO initiated the Vaccine Expedited Review registration procedure for LMICs in 2010, followed by a Collaborative Registration Procedure (CRP) for drugs in 2012 to accelerate local registration of Prequalified products [21]. Through the CRP, WHO provides participating NRAs expanded access to PQ assessment information and in turn NRAs commit to make a registration decision on the products within 90 days.

We are happy to report that scaled up implementation of these efforts by WHO globally and leveraging those initiatives at sub-regional levels as the African Medicines Regulatory Harmonization initiative are currently ongoing and will be will be the subject of publications to follow.

### Limitations

The data presented were collected retrospectively with the support of partners and stakeholders; it did not involve randomization. In addition, the quantitative data available on registration timelines focused mostly on UN Agency-procured products for the treatment or prevention of communicable diseases. Although exhaustive data existed for the WHO PQ program timelines for all prequalified products, timelines for the first SRA registration and for final NRA registration are based on a subset of data kindly provided by multiple partners mentioned earlier. We are, however, confident that these data gaps did not introduce major biases in the analyses, conclusions, and approaches suggested. One of the key take-aways from the effort is that NRAs should further improve transparency, develop tools to monitor their registration timelines to more effectively allocate resources. That would also enable them to identify opportunities for faster access to quality products by the populations under their jurisdictions.

## Conclusion

In conclusion, we believe that the dialogue among stakeholders and partners which was spurred by the information-gathering reported here is a useful step in the efforts to help bring vaccines and medicines to those who badly need them. We have made a data-driven case for optimizing regulatory systems and have proposed collaborative approaches that engage all contributors that we believe can result in meaningful solutions. Implementation of the changes proposed here, some of which are already underway, will primarily benefit products that participate in PQ. In addition, developing efficient, non-redundant, and optimized regulatory systems will benefit all biopharmaceutical products marketed in LMICs. Most importantly, an optimized registration system would move the world closer to help ensure that the future generations of innovative medicines reach the people who could most benefit from them as quickly and efficiently as possible.
